# A Normalized Absolute Values Adaptive Evaluation Function of Image Clarity

**DOI:** 10.3390/s23229017

**Published:** 2023-11-07

**Authors:** Xiaoyi Wang, Tianyang Yao, Mingkang Liu, Kunlei Zheng, Chengxiang Zhao, Longyuan Xiao, Dongjie Zhu

**Affiliations:** 1 School of Mechatornics Engineering, Henan University of Science and Technology, Luoyang 471003, China; yty0101@139.com (T.Y.); kklmk163@163.com (M.L.); zkl15538694420@163.com (K.Z.); zcx_98@126.com (C.Z.); xly15137615751@163.com (L.X.); zdjwsbn@163.com (D.Z.); 2 Henan Key Laboratory of Mechanical Design and Transmission System, Henan University of Science and Technology, Luoyang 471003, China

**Keywords:** clarity evaluation, visual measurement, adaptive background brightness, normalized absolute value

## Abstract

The clarity evaluation function plays a vital role in the autofocus technique. The accuracy and efficiency of the image clarity evaluation function directly affects the accuracy of autofocus and the speed of focusing. However, classical clarity function values are sensitive to changes in background brightness and changes in object contour length. This paper proposes a normalized absolute values adaptive (NAVA) evaluation function of image clarity. It can eliminate the influence of changes in background brightness and the length of the measured object contour on the image clarity function value. To verify the effectiveness of the NAVA function, several experiments were conducted under conditions of virtual master gear images and actual captured images. For actual captured images, the variation of the evaluation results of the NAVA function is far less than the corresponding variation of the classic clarity function. Compared with classical clarity evaluation functions, the NAVA function can provide normalized absolute clarity values. The correlations between the NAVA function results of image clarity and both the contour length and background brightness of the tested object are weak. The use of the NAVA function in automatic and manual focusing systems can further improve focusing efficiency.

## 1. Introduction

In the industrial application of machine vision measurement, the acquisition of a clear image of the object to be measured is a prerequisite for ensuring measurement accuracy [1,2]. In order to improve the degree of automation and measurement efficiency, much attention has been paid to the research and application of autofocus technology [3,4]. Liao et al. [5] proposed a deep learning-based single-shot autofocus method for microscopes applied in life sciences, material research, and automatic detection. Xu et al. [6] proposed a fast autofocus technology based on wavefront sensing, which can improve the efficiency of autofocus. The autofocus technique generally consists of three aspects: clarity evaluation function, focus window selection, and search strategy. Li et al. [7] conducted comparative analysis on some common clarity evaluation functions. Zhu et al. [8] proposed an improved non-uniform sampling method that can improve the accuracy of automatic focusing. Xie et al. [9] proposed an adaptive mountain-climb searching strategy for autofocus system based on No-Reference Structural Sharpness (NRSS) of a single image. The principle of automatic focusing is to select the focusing window and use a search strategy to search for the peak value of the clarity evaluation function curve. The clarity evaluation function plays a crucial role in autofocus technology [10,11]. The accuracy of autofocus and the speed of focusing are directly affected by the accuracy and efficiency of the image clarity evaluation function [12].

Common clarity evaluation functions are generally based on the spatial information, frequency domain information, or informatics information of the image [13,14]. The frequency domain function determines whether the image is clear by analyzing the spectral information obtained by performing Fourier transform on the image. This type of function has high sensitivity, but it requires a large amount of computation and has poor robustness. The informatics function analyzes the clarity of an image by calculating the entropy of the image. The larger the entropy, the richer the information contained in the image, and the higher the clarity. This method generally has poor sensitivity [15]. The spatial function determines whether the image is clear or not according to the drastic degree of change in the gray of the pixel at the edge of the image, and this kind of function has been verified by many practical applications because of its stability, universality, and sensitivity.

In order to improve the performance of autofocus technology, various improved image clarity evaluation functions have been studied in recent years. Zha et al. [16] used a combination of neural networks and classical clarity evaluation functions (such as Brenner, Tenengrad, etc.) to achieve the effect of depth of field expansion. On the basis of blur filtering, Bahy et al. [17] calculated the sum of the absolute values of adjacent pixel grayscale differences in the vertical and horizontal directions, and used the maximum value of the two as the image clarity evaluation index, achieving good results. Lu et al. [18] proposed an autofocus function based on a four neighborhood multi-directional two-level gradient function and a pupil localization function fused with a convolutional neural network intelligent region of interest window. This function significantly improves computational speed compared to classical clarity functions and can be aligned with the measurement optical axis by utilizing the mechanical movement of a three-dimensional high-precision displacement table. Xiong et al. [19] used a combination of Brenner’s function and Roberts’ function to improve the stability and sensitivity of the edge orientation clarity function of micro-nano-structures. Zhou et al. [20] utilized the Tenengrad gradient function to search for the focal position in two steps, which can effectively improve the focusing efficiency and accuracy of the three-axis vision measurement system.

Although the existing clarity evaluation functions have achieved significant improvements in robustness and accuracy in general environments, there are still shortcomings in the existing clarity evaluation functions when facing changes in background brightness and the length of the measured object contour.

The shortcomings of existing functions are as follows: (1) The results of clarity are easily affected by the background brightness. Clarity evaluation results vary with changes in illumination brightness, and there is no uniform standard for clarity of imaging; (2) Clarity evaluation results are susceptible to the contour length of the object being measured, due to lack of uniform evaluation criteria for the clarity of images for different shapes of objects.

The principle of autofocus is to search for the peak of the clarity evaluation function curve using a search strategy after selecting the focus window. When the background brightness of the tested object image changes and the contour length of the tested object changes, the clarity value calculated by the existing clarity evaluation function will change, resulting in a change in the maximum image clarity value, that is, the peak and position of the clarity curve will change. At this point, the autofocus algorithm requires repeated attempts to find the clearest position, resulting in longer autofocus time and lower efficiency.

To address the above problems, an adaptive evaluation method for normalized absolute values of image clarity, called NAVA (Normalized Absolute Values Adaptive) method is proposed. This method calculates clarity based on the characteristics of the image in the spatial domain: (1) By monitoring and compensating for background brightness, the influence of background brightness changes on clarity evaluation results can be reduced; (2) By selecting the grayscale difference among a limited number of pixels from the image contour length and using the sum as a function value, the influence of object contour length on the clarity evaluation results can be reduced. We conducted experiments on changing background brightness, on changing the contour length of the measured object, and on two objects with different object distances. The experimental results demonstrate that this method can significantly improve the efficiency of autofocus and shorten the time required for autofocus.

The structure of this paper is as follows. In Section 2, conventional spatial functions are compared and analyzed, and the definition and calculation process of the NAVA function are provided. In Section 3, we present results of experiments that were conducted on changes in background brightness, contour length, and the presence of two different object distances in the field of view. The experiments were conducted using virtual master gear images and actual captured images. The experimental data verified the effectiveness of the NAVA function. Section 4 is the conclusion.

## 2. Principle of the Clarity Evaluation Function

In visual measurement, the clearer the image, i.e., the sharper the edges of the image, the greater the grayscale difference between adjacent pixels. By utilizing this feature, a series of classical and improved functions for evaluating image clarity are derived by combining different point selection rules and operation methods.

The classical image clarity evaluation functions include the Brenner function, Roberts function, Energy of Gradient (EOG) function, and so on [21].

However, when the background brightness of the image or the contour length of the object to be measured changes, the classical clarity evaluation function mentioned above cannot provide a fixed and consistent evaluation result.

The NAVA function proposed in this article can provide normalized absolute values of image clarity. When the background brightness and contour length change, the NAVA clarity evaluation value hardly changes.

### 2.1. Classical Clarity Evaluation Functions

Brenner, Roberts, and EOG are classical clarity functions that have the advantage of being simple in principle and easy to compute. The formula for the clarity evaluation values of the functions are as follows.

Brenner function:

(1) $$F=\sum_{x=1}^{M-2}\sum_{y=1}^{N}{[I(x+2,y)-I(x,y)]}^{2}$$

The Brenner function uses the sum of the squares of the grayscale differences between pixels separated by two pixels as the evaluation value for image clarity.

Roberts function:

(2) $$F=\sum_{x=1}^{M-1}\sum_{y=1}^{N-1}\{[I(x+1,y+1)-I(x,y){]}^{2}+[I(x+1,y)-I(x,y+1){]}^{2}\}$$

The Roberts function utilizes the cumulative value of the sum of the squares of the gray differences between two adjacent pixel points on a ±45° diagonal as the clarity evaluation value.

EOG function:

(3) $$F=\sum_{x=1}^{M-1}\sum_{y=1}^{N-1}\{[I(x+1,y)-I(x,y){]}^{2}+[I(x,y+1)-I(x,y){]}^{2}\}$$

The EOG function uses the cumulative value of the sum of the squares of the gray differences between two adjacent pixel points in the horizontal and vertical directions as the clarity evaluation value. 

In the formulas, *M* and *N* are the number of rows and columns of the image pixels, respectively.

The following conclusions can be drawn from the formulas of the Brenner function, Roberts function, and EOG function:

(1) The clarity evaluation value is related to the background brightness of the image. When the background brightness changes, the clarity evaluation value will also change accordingly; (2) The clarity evaluation is related to the length of the edge contour of the object being measured. The longer the contour length, the greater the calculated clarity value.

As a result, the peaks of the clarity values obtained using clarity evaluation functions such as Brenner, Roberts, and EOG change when the brightness of the image background changes and when the object to be measured is replaced (resulting in a change in the length of the edge contour). When these conventional sharpness evaluation functions are used for autofocus, frequent calibration is usually required to obtain more accurate sharpness peaks, resulting in longer time consumption and lower efficiency.

### 2.2. NAVA Function

In order to improve the focusing efficiency of machine vision measuring instruments such as video measuring machines and industrial microscopes, a clarity evaluation function called NAVA, which is almost unaffected by background brightness and image contour length, has been constructed. The first part is to calculate the peak image clarity *G_max_* and the background grayscale value *G_back0_* corresponding to the peak, which is called calibration.

**Figure 1:**
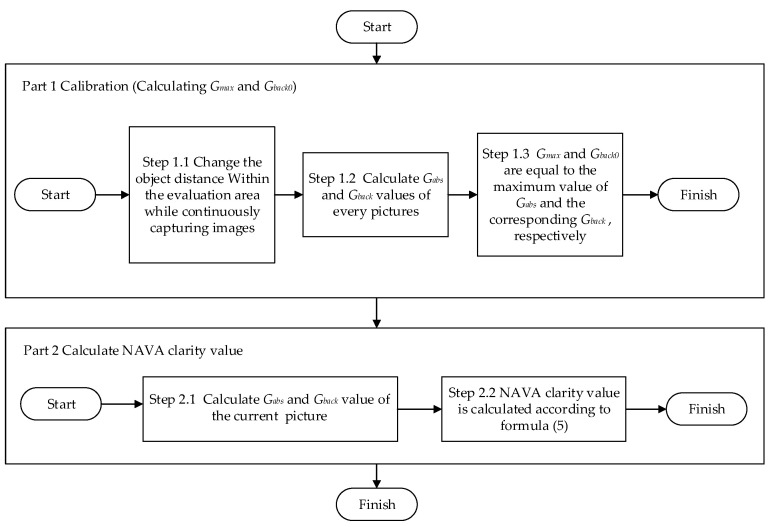
Flowchart of NAVA function to calculate image clarity.

Secondly, after completing the calibration, calculate the output value of the clarity function for any image obtained. The flowchart of calculating image clarity using the NAVA function is shown in Figure 1.

As shown in Figure 1, the calibration process of the NAVA clarity function consists of the following three steps. Firstly, move the camera or object to be tested from its initial position to its final position (i.e., change the object distance), while maintaining the brightness of the background lighting and continuously capturing multiple images. Secondly, calculate the *G_abs_* and *G_back_* values of all captured images. Thirdly, select the maximum value from all *G_abs_* values as *G_max_*, and record the background grayscale value *G_back_* of the image corresponding to *G_max_* as *G_back0_*.

In the above steps, *G_abs_* is the absolute value of image clarity, which is used to eliminate the influence of contour length on the clarity evaluation results. *G_back_* is the grayscale value of the image background, which is used to eliminate the influence of background brightness changes. The calculation method of *G_abs_* is as follows. Assume the image resolution is *M* × *N*. First, starting from the first pixel in the *i*-th row, calculate the grayscale difference between the two pixels separated by m in that row, obtain the absolute values of all *N* – *m* − 1 grayscale differences, and find the maximum value among them, denoted as *G_imax_*. Next, calculate the *G_imax_* values of all rows and arrange them in descending order. Finally, from the arranged *G_imax_* array, select the values in the range from the top P% to Q% and sum them. This sum is used as an indicator to evaluate the absolute clarity of the image, denoted as *G_abs_*. The method for calculating *G_imax_* is shown in Formula (4):

(4) $$G_{\mathrm{imax}}=\max(|G(i,j)-G(i,j+m+1)|j=1,2,3,\cdots ,N-m-1)$$

In the formula, *G_(i, j)_* represents the grayscale value of the *i*-th row and *j*-th pixel in the image.

Here, the parameters m, P, and Q need to be reasonably selected based on the actual image acquisition system in order to enable the NAVA function to better perform its functions. Among them, changing the value of m will affect the sensitivity of the function. The value of m should be chosen as large as possible while being smaller than the width of the blurred area at the edge of the image. The purpose of introducing P and Q values is to increase the robustness of the algorithm. For systems with more noise, the P value should be higher and the Q value should be lower. The resolution of the image acquisition system is 2456 × 2058 pixels, and the values of m, P, and Q can be set to 10, 5, and 10.

**Figure 2:**
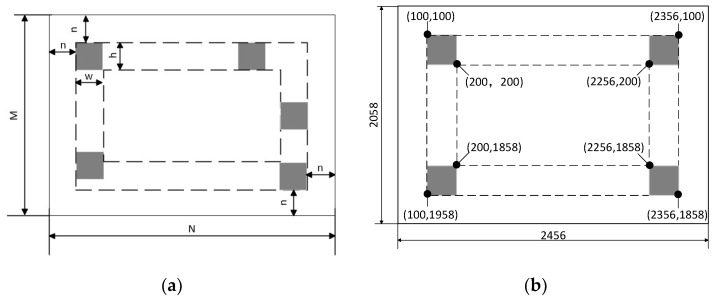
Background brightness monitoring. (**a**) Background brightness monitoring scheme; (**b**) Example of background brightness monitoring area.

In the NAVA function, the image background brightness value *G_back_* can be calculated using multiple methods. One of the methods is to set multiple background brightness monitoring areas with height and width of h and w in the edge area of the image, with a distance of n pixels from the edge, as shown in Figure 2a. These background brightness monitoring areas should avoid the measured objects in the image. Calculate the average of the grayscale values of all background brightness monitoring areas and sort them. Take the grayscale average of the monitoring area corresponding to the median as the background brightness value *G_back_* of the current image. For the images in the experiment, four background brightness monitoring areas can be set up with n, h, and w equal to 100 (see Figure 2b), which can achieve better results.

After the calibration is completed, *G_max_* and *G_back0_* are obtained, and the clarity of a newly captured image can be calculated. The calculation process includes the following steps. Firstly, calculate the *G_abs_* and *G_back_* values of the newly collected image (current image). Secondly, calculate the clarity percentage according to Formula (5) as the output value of the NAVA function:

(5) $$\mathrm{NAVA}=\frac{G_{\mathrm{abs}}\times G_{\mathrm{back}0}}{G_{\max}\times G_{\mathrm{back}}}\times 100\%$$

The key for the NAVA function to resist the changes in image background brightness and the length of the measured object’s contour is to normalize the length of the contour and the image background brightness. After normalization, when there are multiple objects of different distances and sizes in the feasible area (i.e., the range of object distance variation) of autofocus, the values given by the NAVA function in the clearest position of each object are almost the same, which can greatly improve the efficiency of focusing on multiple objects in the field of view.

## 3. Testing and Analysis

**Figure 3:**
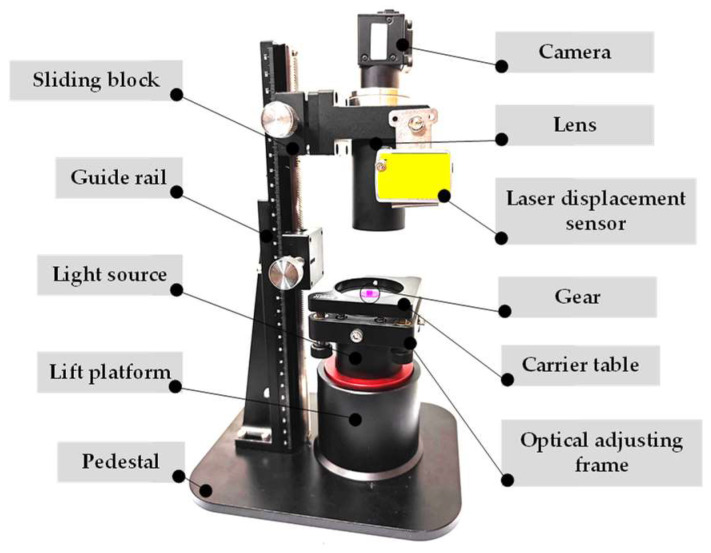
Test platform.

The test platform consists of a camera, a lens, a guide rail, a slider, a laser displacement sensor, a light source, an optical adjustment frame, a lift platform, and a base, as shown in Figure 3. In this research, fine-pitch gears were selected as the test object [22]. In order to reduce the influence of other factors on the clarity of the image in the test, a monochromatic LED light source was selected, and the level of the carrier table was calibrated before the test by means of an optical adjustment frame. The tests were conducted on a personal computer equipped with a 12th Gen Intel(R) Core(TM)i5-1240 processor and an Intel(R) UHD Graphics 730 GPU.

In addition to using the gear images collected by the camera to verify the actual effect of the clarity function, virtual gear artifacts can also be used to verify the theoretical effect of the clarity function. The virtual master gear is an error-free gear image generated by a program that has the same or similar features as the measured image of the actual artefact, which can be used to verify the correctness and robustness of the clarity function. Compared to using actual gear images, testing the clarity function using virtual master gear images has the following advantages: (1) There will be no pixel defects in the camera sensor; (2) There will be no image distortion caused by the lens; (3) No sensor noise problems will occur; (4) There will be no dust or fibers that interfere with the test results.

In order to verify the effectiveness of the NAVA clarity function, three experiments were designed, namely, (1) an experiment to change the background brightness, (2) an experiment to change the length of the measured object’s contour, and (3) an experiment to simultaneously place multiple objects of different heights. These three experiments were conducted using virtual master gear images and actual gear images.

### 3.1. Experiment on Changing Background Brightness

**Figure 4:**
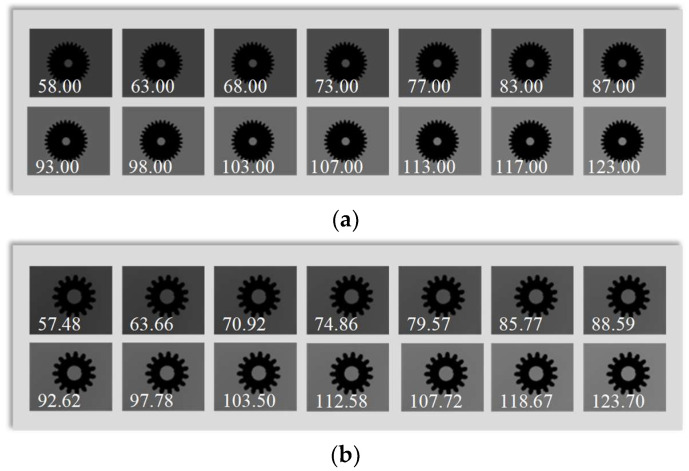
Gear images with different background brightness (numbers in the images are background gray values). (**a**) Images of virtual master gear; (**b**) Images of actual gears.

In the experiment, first adjust the object distance until the image of the tested object is clear. Then, keep the object distance unchanged and change the brightness of the background. Set 14 levels of background brightness within the feasible range and capture images separately. Correspondingly, generate 14 virtual gear images with different background brightness. The virtual master gear images are shown in Figure 4a and the actual gear images are shown in Figure 4b. The pixel resolution of both actual and virtual images is 2456 × 2058.

**Figure 5:**
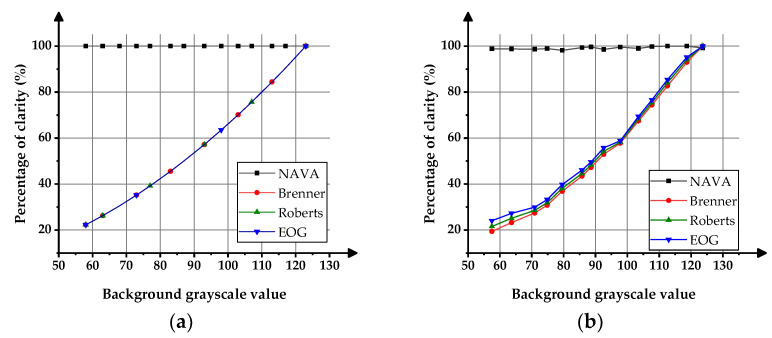
Effects of background brightness variation on the result of clarity functions. (**a**) Experiment with virtual gear images; (**b**) Experiment with actual gear images.

Figure 5 shows the influences of background brightness changes on the clarity functions. In the figure, the horizontal scale represents the background grayscale, and the vertical scale represents the normalized calculation results of various clarity functions. In the experiment, the NAVA function, Brenner function, Roberts function, and EOG function were used to calculate the clarity of virtual gear images and actual gear images with different background brightness.

The foresaid four different clarity functions have significant differences in the absolute values of clarity calculated from the same image.

In order to compare the stability of the functions, it is necessary to normalize the calculation results of various clarity functions. That is, for each of the clarity functions, calculate the clarity values of 14 images and find the maximum among them. Then, divide the clarity value of each image by the maximum clarity value, calculate the percentage as the evaluation result, and draw it on the same image for comparison. The clarity evaluation results of the virtual master gear images are shown in Figure 5a, and the clarity evaluation results of the actual gear images are shown in Figure 5b.

It can be observed from Figure 5 that the clarity evaluation results of the NAVA function remain stable in both virtual image experiments and actual image experiments, and are almost unaffected by changes in background brightness. However, classical Brenner, Roberts, and EOG functions lack compensation for changes in light source brightness, resulting in significant changes in clarity evaluation results as the background gray value increases.

**Figure 6:**
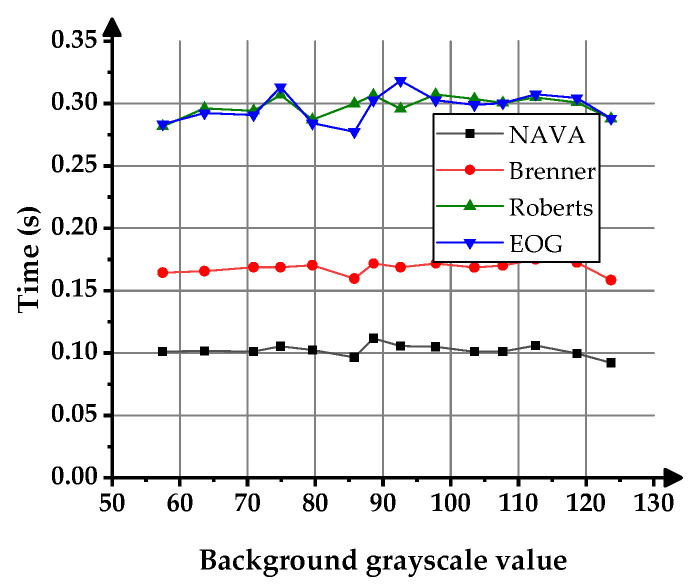
Time taken for calculating image clarity results using the clarity evaluation function.

In the figure, the horizontal scale represents the background grayscale, and the vertical scale represents the time taken by various clarity functions to calculate the image clarity results. Figure 6 shows the time taken by the NAVA function, Brenner function, Roberts function, and EOG function to calculate the 14 images shown in Figure 4b. It can be seen that the calculation speed of the NAVA function is faster than the other three functions when calculating the same image in the same environment. Due to the fact that the duration of calculating images using sharpness algorithms has the same trend as the influence of images, subsequent experiments will no longer display the time information of each algorithm’s calculation of corresponding images one by one.

Therefore, in some application scenarios where the background brightness may change frequently, using the NAVA function can obtain more stable clarity calculation results than using classical functions, better maintain the focus state, and achieve higher focus efficiency, thereby producing a better user experience.

### 3.2. Experiment on Changing the Contour Length of the Measured Object

In the experiment, 14 virtual gear images and 10 actual gear images of different sizes were used, while the background brightness and object distance remained unchanged.

**Figure 7:**
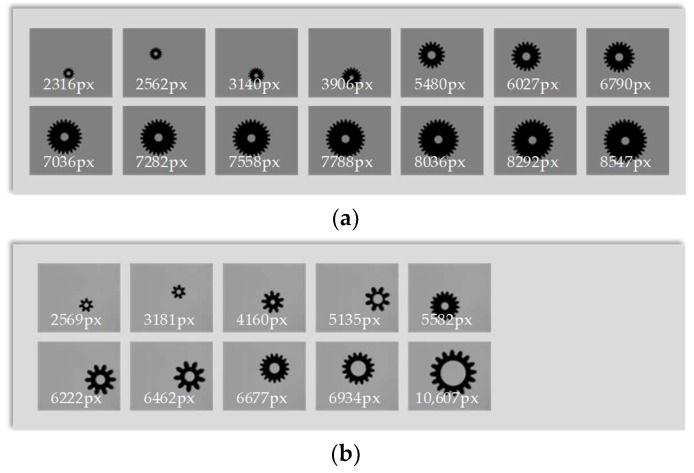
Images of gears with different contour lengths (numbers in the images are contour length in pixels). (**a**) Images of virtual master gears; (**b**) Images of actual gears.

The virtual master gear images are shown in Figure 7a and the actual gear images are shown in Figure 7b. We used NAVA function, Brenner function, Roberts function, and EOG function to calculate the clarity values.

**Figure 8:**
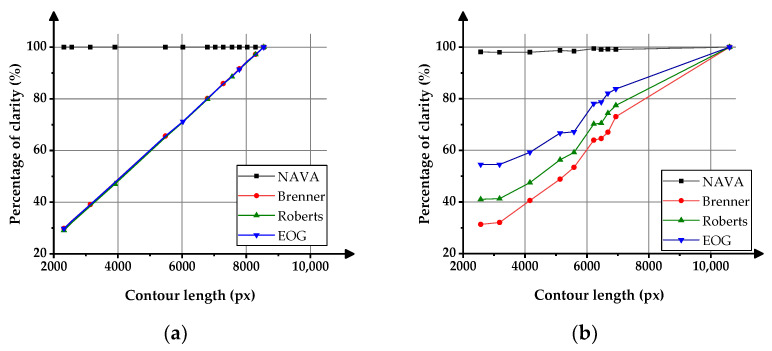
Effects of contour length variation on clarity function values. (**a**) Experiment of virtual gear images; (**b**) Experiment of actual gear images.

Figure 8 shows the effects of contour length changes on the clarity values. In the figure, the horizontal scale represents the contour length of the gears in pixels, and the vertical scale represents the percentages of image clarity. The variation curve of the clarity function value of virtual master gear images is shown in Figure 8a, and the variation curve of the clarity evaluation function value of the actual gear images is shown in Figure 8b.

In Figure 8a, the clarity evaluation results of the NAVA function remain unchanged when the contour length of the virtual master gear changes, proving that the image clarity evaluation value of the NAVA function is theoretically not affected by the contour length of the tested gear. In Figure 8b, when the length of the gear contour changes in the actual gear photo taken, the value of the NAVA function changes slightly, and the change magnitude is much smaller than other clarity functions.

Further analysis suggests that the reason for the slight change in the clarity evaluation results of the NAVA function in Figure 8b may be that, when taking actual photos with the camera, there are changes in the brightness of the light source and the ambient lighting, which can cause slight differences in the background grayscale value of the photo and slightly affect the value of the NAVA function. Actual gears of different sizes have different heights, making it difficult to ensure that the actual clarity of each gear is exactly the same during manual focusing in the experiment.

As seen from the experimental data, the variation in the clarity evaluation results of the NAVA function is within 3%. In contrast, the Brenner function, Roberts function, and EOG function are greatly affected by changes in contour length, with variations exceeding 60%.

Therefore, when replacing objects with different contour lengths, it is necessary to recalibrate the peak of the clarity evaluation curve when using Brenner, Roberts, and EOG functions. When using the NAVA function, there is no need to search for the peak of the sharpness curve again, which can improve the efficiency of autofocus.

### 3.3. Experiment on Two Objects with Different Object Distances

The NAVA function can provide normalized absolute clarity evaluation results for the measured objects at different distances. To verify this feature, two gears with different facewidths and contour lengths were placed on the carrier table. During the movement of the carrier table, images were captured at 20 different distances.

**Figure 9:**
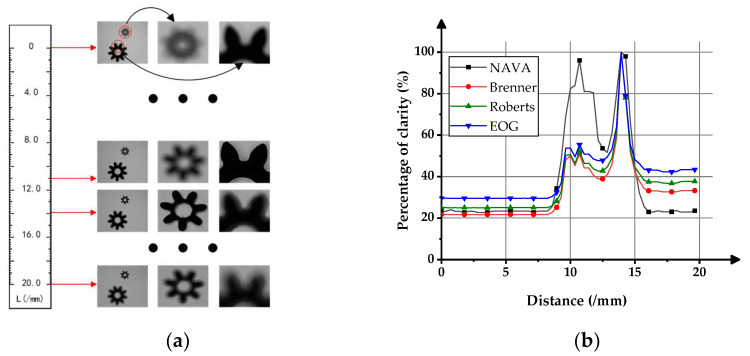
Clarity evaluation experiment of placing two objects simultaneously. (**a**) Images of actual gears captured at different distances; (**b**) The curve of clarity evaluation values changing with object distance.

Figure 9a shows the actual gear image taken. Theoretically, multiple objects with different object distances can be placed simultaneously within the camera’s field of view, but, due to the limitation of the size of the gears used in this experiment, only two gears can be placed.

Figure 9b shows the curves of four different clarity evaluation functions changing with object distance. The horizontal scale represents the object distance, and the vertical scale represents the percentage of clarity.

In the experiment, the clarity function achieved maximum values at object distances of 11 mm and 13.9 mm. Among them, the NAVA function gave clarity evaluation results of 96.60% and 100% at the two clearest positions, respectively. Brenner’s clarity evaluation results were 51.02% and 100%, respectively. Roberts’ clarity evaluation results were 52.38% and 100%, respectively. The clarity evaluation results of EOG were 55.38% and 100%, respectively. Compared with the other three functions, the evaluation results of NAVA function can better assist operators or automated systems in finding the optimal shooting distance for objects at different distances.

## 4. Conclusions and Future Work

In order to solve the problem that the calculation results of classical clarity functions vary significantly with the background brightness of the image and the length of contour of the measured object, an adaptive clarity evaluation function called NAVA is proposed. In order to verify the effectiveness of the NAVA function in resisting interference from background brightness and changes in the contour length of the tested object, virtual master gear images and actual captured images were used to conduct a background brightness change experiment, contour length change experiment, and experiment of two tested objects with two different object distances. The experimental data show that the clarity evaluation results of the NAVA function under the conditions of virtual master gear images are completely unaffected by changes in the background brightness and the length of the object contour, which theoretically verifies the feasibility of the NAVA function. Under the conditions of actual captured gear images, the NAVA function result changes less than 2% in the background brightness change test, changes less than 3% in the contour length change test, and the image clarity evaluation results of the two objects to be tested in the double-object test are 100% and 96.60%. This means that the fluctuation of the NAVA function is much smaller than that in the conventional clarity functions.

Compared with the classical clarity evaluation functions, the NAVA function can effectively resist the influence of background brightness changes and the contour length changes of the tested object. Based on these two characteristics, the NAVA function has the following advantages: (1) The influence of changes in lighting brightness on the clarity evaluation value of images is relatively small, which can reduce the influence of changes in background brightness caused by natural light or light source voltage changes in the actual measurement environment; (2) The change in the contour length of the measured object has little influence on the image clarity evaluation value. After completing a peak clarity calibration of the given measurement system, a more accurate clarity evaluation can be made for the measured objects with different contour lengths; (3) It is possible to provide fair clarity evaluation results for multiple objects with different object distances within the camera’s field of view, improving the focusing efficiency of the camera. Machine vision inspection technology has become an important tool in smart manufacturing and smart inspection, and the NAVA function proposed in this paper can play an important role in autofocus technology.

However, in order to achieve the goal of not being affected by changes in contour length in the clarity evaluation function, the NAVA function made trade-offs, reducing the number of pixels involved in clarity evaluation, which would make the function value more sensitive to local image noise. Although methods such as selecting the gray values of special interval pixels as clarity evaluation values can filter out some noise, the problem of small fluctuations in clarity evaluation results caused by the influence of noise on the NAVA function still exists. Our next consideration is how to reduce the effect of noise is the key to subsequently improve the stability of the NAVA function.

## Data Availability

The data that support the findings of this study are available from the corresponding author upon reasonable request.

## References

[1] Lu R., Wu A., Zhang T., Wang Y. (2018). Review on automated optical (visual) inspection and its applications in defect detection. Acta Optica Sinica.

[2] Qi K., Shen P., Chen J., Wang H. (2023). Image Definition Evaluation Method Based on Edge Feature. Mach. Des. Manuf..

[3] Ye S., Zhong W., Qu X. (2000). Review and Prospect of Precision Inspection. China Mech. Eng..

[4] Shi Z., Fang Y., Wang X. (2022). Research Progress in Gear Machine Vision Inspection Instrument and Technology. Laser Optoelectron. Prog..

[5] Liao J., Chen X., Ding G., Dong P., Ye H., Wang H., Zhang Y., Yao J. (2022). Deep learning-based single-shot autofocus method for digital microscopy. Biomed. Opt. Express.

[6] Xu J., Gao S., Jiang Z., Kong Y., Liu C., Wang S. (2020). Wavefront sensing based autofocus method and its applications. J. Optoelectron. Laser.

[7] Li Z., Li X., Ma L., Hu Y., Tang L. (2011). Research of Definition Assessment based on No-reference Digital Image Quality. Remote Sens. Technol. Appl..

[8] Zhu K., Jiang W., Gao Z., Zhou X., Zhang J. (2006). Focusing Window Choice and Parameters Determination in Automatic Focusing System. Acta Optica Sinica.

[9] Xie X., Zhou J., Wu Q. (2011). An Adaptive Autofocus Method Using No-reference Structural Sharpness. Opto-Electron. Eng..

[10] Liu S., Liu M., Yang Z. (2016). An image auto-focusing algorithm for industrial image measurement. EURASIP J. Adv. Signal Process..

[11] Jin S. (2022). Optical Design and Verification of Multi-Layer Rapid Autofocus System for Microscopic Systems. Bachelor’s Thesis.

[12] Caviedes J., Oberti F. (2004). A new sharpness metric based on local kurtosis, edge and energy information. Signal Process. Image Commun..

[13] Wang Y., Feng H., Xu Z., Li Q., Chen Y. (2016). Autofocus Evaluation Function Based on Saturate Pixels Removing. Acta Optica Sinica.

[14] Zhang L. (2016). Research of Image Sharpness Assessment Algorithm for Autofocus. Bachelor’s Thesis.

[15] Liu J., Lu R., Zhang Z., Zhang A. (2023). Sharpness Evaluation Function for Line Patterns in Focal Length Measurement. Acta Optica Sinica.

[16] Cha Z. (2022). Research on Technologies of Auto-Focus and Multi-Focus Fusion for Medical Ultre HD Camera System. Bachelor’s Thesis.

[17] Bahy R.M. (2021). Autofocus microscope system based on blur measurement approach. J. Physics. Conf. Ser..

[18] Wang L., Gong Y., Zhang Y., Lang S., Zheng H. (2023). Human Eye-Autofocus and Pupil Center Auto-Alignment system. Acta Optica Sinica.

[19] Xiong R., Gu N., Xu H. (2022). An Auto-Focusing Evaluation Function Adapted to Multi-Directional Gray Gradient Change. Laser Optoelectron. Prog..

[20] Zhou P., Hu C., Bi C., Hao X. (2021). Auto focusing technology of three-axis vision measuring system. Infrared Laser Eng..

[21] Ao X., Liu C., Zhang D. (2023). EOG-DS: Zoom Dual-Mode Tracking Algorithm for Dynamic Targets. Comput. Eng. Appl..

[22] Zhi S., Zhao W., Zhao W., Duan Z., Sun H. (2018). Visual measurement method of pitch machine based on gear local image. Chin. J. Sci. Instrum..

